# Have We Forgotten the Threat Posed by HIV?

**Published:** 2018-07

**Authors:** Małgorzata PAJĄCZEK, Tatyana HALYTSKA, Tomasz KRYCZKA

**Affiliations:** 1. National Health Fund Central Office, Warsaw, Poland; 2. Dept. of Medical Biology, Medical University of Warsaw, Warsaw, Poland

## Dear Editor-in-Chief

We have read with great interest the paper published in Iranian Journal of Public Health in Sep 2016 (1). The authors aimed to evaluate the epidemiologic research priorities in the field of HIV and AIDS in Iran. According to their data between 1996 and 2014, 362 epidemiologic studies were conducted in Iran involving 10,122,239 participants. In that paper, the authors discussed the former areas of research and indicated further research topics regarding the Iranian population, in response to a changing epidemiological situation in the country. The conclusions of that review prompted us to reconsider our knowledge about current epidemiology status of HIV and AIDS in Poland as well as European Union (EU).

Among the countries bordering with European Union, Ukraine has one of the highest HIV prevalence in Eastern Europe. Within the very short time, from 2005 to 2012, the incidence of new HIV infections increased from 29.1 to 45.5, morbidity of AIDS – from 8.9 to 22.1 and prevalence of HIV in Ukraine increased from 133.5 to 283.4 per 100,000 inhabitants (2). Significantly, in reference to Ukraine, the highest HIV incidence was noted in the eastern provinces (oblasts) bordering with Russia (3). The political crisis and military invasion from Russia, which Ukraine faced in 2014, let to the increased migration of Ukraine citizens from the Eastern part of Ukraine to the Western one, and further – to the EU. The main route of this migration of Ukrainians to the EU led through Poland, the closest EU-country. Unfortunately, nobody precisely knows how many Ukrainians have arrived in Poland so far. There are the Ukrainian asylum seekers and the ones applying for temporary or permanent residence in Poland (Table 1), but the most reliable data in the subject are the number of registrations of the Ukrainian citizens for work in Poland. In 2015 as a whole, 762,700 statements of intent to entrust a job to a Ukrainian citizen were registered and in the first half of 2016 – an additional 614,196 (4). Therefore, the general estimations of the authorities vary between 800,000 and 1.5–2 million Ukrainians working in Poland or EU.

**Table 1:** Ukrainian citizens applying to the Polish authorities in the recent years

***YEAR***	***2007***	***2008***	***2009***	***2010***	***2011***	***2012***	***2013***	***2014***	***2015***
group A	55	40	36	45	67	72	46	2,318	2,305
group B	8,558	9,054	9,609	9,844	9,114	11,743	11,111	23,390	58,740

group A: number of Ukrainian asylum seekers applying in Poland; group B: number of Ukrainian citizens applying for temporary or permanent residence in Poland (4)

At the moment all medical actions related to the immigrants, refugees and asylum seekers are based on the local EU-states regulations. For example, in Poland, the chest x-rays and taking the blood samples are the part of so-called ‘epidemiological filter’. Concerning blood samples the following parameters are measured, e.g., basic blood cells counts and biochemistry tests enriched with immunology tests toward e.g. HBV, HCV, tuberculosis, and HIV. Within the years 2008–2015 about 39,000 asylum seekers (not only from Ukraine) were screened with these tests in Poland and 303 of them were revealed HIV positive (5). These data are not impressive but it should be taken into consideration that this number of HIV patients was discovered only in the very narrow group of immigrants – the asylum seekers. Meanwhile, there are no epidemiological data on the remaining Ukrainians – up to 2 million citizens (4) – that stayed in Poland or passed the country heading to EU. Unfortunately, this huge group of immigrants is not covered by the regulations described above, as only the minority of Ukrainians applied for asylum in Poland. For the remaining immigrants, the ‘Act on Foreigners’ of Dec 12, 2013 (6) regulates their access to the public healthcare service, but none of the articles of this regulation treats about diagnostic procedures toward infectious diseases in this group of the incomers. Unfortunately, the most of the EU-states face the same situation that leads to the disagreeable thought – HIV epidemics is a ticking time bomb. Not only due to the number of Ukrainians in Europe, but also due to the increased flow of migrants and refugees from all directions to the European Union.
